# Activation of Vitamin D Regulates Response of Human Bronchial Epithelial Cells to *Aspergillus fumigatus* in an Autocrine Fashion

**DOI:** 10.1155/2015/208491

**Published:** 2015-04-15

**Authors:** Pei Li, Ting Wu, Xin Su, Yi Shi

**Affiliations:** Department of Respiratory and Critical Care Medicine, Jinling Hospital, Nanjing University School of Medicine, 305 East Zhongshan Road, Nanjing, Jiangsu 210002, China

## Abstract

*Aspergillus fumigatus* (*A. fumigatus*) is one of the most common fungi to cause diseases in humans. Recent evidence has demonstrated that airway epithelial cells play an important role in combating *A. fumigatus* through inflammatory responses. Human airway epithelial cells have been proven to synthesize the active vitamin D, which plays a key role in regulating inflammation. The present study was conducted to investigate the impact of *A. fumigatus* infection on the activation of vitamin D and the role of vitamin D activation in *A. fumigatus*-elicited antifungal immunity in normal human airway epithelial cells. We found that *A. fumigatus* swollen conidia (SC) induced the expression of 1*α*-hydroxylase, the enzyme catalyzing the synthesis of active vitamin D, and vitamin D receptor (VDR) in 16HBE cells and led to increased local generation of active vitamin D. Locally activated vitamin D amplified SC-induced expression of antimicrobial peptides in 16HBE cells but attenuated SC-induced production of cytokines in an autocrine fashion. Furthermore, we identified *β*-glucan, the major *A. fumigatus* cell wall component, as the causative agent for upregulation of 1*α*-hydroxylase and VDR in 16HBE cells. Therefore, activation of vitamin D is inducible and provides a bidirectional regulation of the responses to *A. fumigatus* in 16HBE cells.

## 1. Introduction

*Aspergillus fumigatus* (*A. fumigatus*) is ubiquitous, saprophytic, and airborne mold that causes a range of lung diseases in humans [1, 2]. Airway epithelial cells serve as the first line of the host defense system against* A. fumigatus*. Recognition of* A. fumigatus* by airway epithelial cells results in gene transcription and secretion of a variety of effector molecules, including antimicrobials, type I interferons, and proinflammatory cytokines and chemokines, suggesting that these cells play an important role in combating inhaled* A. fumigatus* infection through inflammatory responses [3–8]. Meanwhile, airway epithelial cells have a key role in maintaining the inflammatory homeostasis in order to keep gas-exchange surface integrity and stretch the discriminatory powers of the immune system to its limits [9]. As exposure to inhaled* A. fumigatus* conidia is common and lifelong [10], and hosts with normal immunity rarely develop pulmonary aspergillosis, we were curious about how airway epithelial cells maintain the inflammatory balance when interacting with* A. fumigatus* in the lungs of healthy hosts.

Vitamin D, a pluripotent hormone whose functions extend beyond its classical role in calcium homeostasis, has recently been recognized as an important modulator of the pulmonary defense and inflammatory processes [11–15]. The active form of vitamin D, 1,25-dihydroxyvitamin D_3_ (1,25D_3_), has been shown to decrease the production of respiratory syncytial virus-induced NF-kappaB-linked chemokines and cytokines [16] and to inhibit proinflammatory cytokine release from LPS stimulated cystic fibrosis (CF) respiratory epithelial cells [17]. Furthermore, 1,25D_3_ has been shown to induce antimicrobial actions in epithelial cells by virtue of its ability to upregulate expression and secretion of the antimicrobial peptide LL-37 [17–21]. Thus, vitamin D may dampen the inflammatory response to pathogens without negatively affecting pathogens clearance. The biological effects of vitamin D are achieved through the regulation of gene expression mediated by the vitamin D receptor (VDR), which is expressed in virtually all cells in the body [11, 22]. An increasing variety of tissues and cell types have been found to express 1*α*-hydroxylase, the enzyme responsible for the final and rate-limiting step of active vitamin D synthesis, in which 25-hydroxyvitamin D_3_ (25D_3_), the primary circulating or storage form of vitamin D, is converted to 1,25D_3_ [19, 23–28]. Local synthesis of tissue-specific active vitamin D rather than systemic production is thought to be responsible for the immunomodulatory effects of vitamin D. Human airway epithelial cells have also been proven to express 1*α*-hydroxylase [19], indicating that active vitamin D can be produced locally within the airway epithelium, which may act in an autocrine or paracrine fashion to modulate airway immune function.

Vitamin D deficiency is highly prevalent and has been associated with increased susceptibility to respiratory infections, including ABPA [29–40]. In our previous study, we found that vitamin D deficiency caused an aggravated inflammatory response and an impaired host defense to pulmonary challenge with* A. fumigatus* in immunocompetent mice [41]. Based on the fact that local synthesis of active vitamin D occurs in airway epithelium, we investigated the role of* A. fumigatus* in modulating the expression of 1*α*-hydroxylase and VDR in normal human airway epithelial cells and how this affects antifungal immunity in the airway epithelium.

## 2. Materials and Methods

### 2.1. *A. fumigatus* Strain and Preparation of Conidia

The* A. fumigatus* strain was obtained from a fatal case of pulmonary aspergillosis at the Department of Respiratory and Critical Care Medicine, Jinling Hospital, Nanjing University School of Medicine. Conidia were harvested by washing a 7-day-old slant culture on Sabouraud dextrose agar (10 g/L peptone, 40 g/L glucose, and 15 g/L agar) with phosphate-buffered saline (PBS) supplemented with 0.1% Tween 20. The suspension was filtered through a 40 *μ*m cell strainer (Falcon) to separate conidia from the contaminating mycelia, and the absence of mycelia in the filtrate was verified microscopically. Inactivated resting or swollen conidia were obtained as described previously [42, 43]. Resting conidia (RC) were washed with PBS three times and counted. For experiments with swollen conidia (SC), conidia were incubated for 3.5 h in RPMI 1640 (Invitrogen Gibco, Carlsbad, CA, USA) at 37°C. Since human cells have to be exposed to the different forms of* A. fumigatus* for various periods of time (including 24 hours to allow the RC to swell, germinate, and grow into hyphae), all* A. fumigatus* morphotypes were heat-inactivated at 90°C for 60 min.

### 2.2. Cell Line and Growth Conditions

16HBE cells were originally isolated from human bronchial epithelial cells and transformed with the origin-defective simian virus 40 genome. The 16HBE cells used in this study were kindly provided by Professor Laiyu Liu (Department of Respiratory Diseases, Nanfang Hospital, Southern Medical University, Guangzhou, China). 16HBE cultures were maintained in high glucose Dulbecco's Modified Eagle's Medium (DMEM; Invitrogen Gibco) containing 10% fetal bovine serum (FBS; Invitrogen Gibco), 2 nM L-glutamine, 100 U/mL penicillin, and 100 *μ*g/mL streptomycin at 37°C in a humidified 5% CO_2_ atmosphere.

### 2.3. Cell Treatments

16HBE cells were treated with RC or SC (multiplicity of infection (MOI) = 1-2), or *β*-glucan (Sigma-Aldrich, St. Louis, MO, USA) in the absence or presence of inactive vitamin D (25D_3_; Sigma-Aldrich) or active vitamin D (1,25D_3_; Sigma-Aldrich) for appropriate durations. The concentrations of *β*-glucan, 25D_3,_ and 1,25D_3_ used as well as the treatment durations are detailed in the figure legends.

### 2.4. Western Blot Analysis

Cells were lysed with a 1% NP-40 hypotonic lysis buffer containing 1 mM phenylmethanesulfonyl fluoride, 1% aprotinin, and 1 mM sodium vanadate (Sigma-Aldrich). Cell lysates were mixed with loading buffer, boiled for 5 min, resolved by 12% sodium dodecyl sulfate-polyacrylamide gel electrophoresis, and transferred onto a polyvinylidene fluoride membrane (Millipore, Bedford, MA, USA). This membrane was subsequently blocked with 5% nonfat milk. Western blot analysis was then performed in accordance with standard protocols using antibodies (Santa Cruz Biotechnology, Santa Cruz, CA, USA) against 1*α*-hydroxylase, VDR, LL-37, or *β*-defensin-2 (HBD2), followed by incubation with relevant horseradish peroxidase-conjugated secondary antibodies (Santa Cruz Biotechnology). Relative changes in protein expression were estimated from the mean pixel density using Quantity One software 4.6.2 (Bio-Rad, Hercules, CA, USA), normalized to glyceraldehyde-3-phosphate dehydrogenase (GAPDH), and presented as relative density units.

### 2.5. Quantitative Real-Time Polymerase Chain Reaction (PCR)

Total RNA was isolated from cultured cells, and cDNA synthesis was performed on the RevertAid First Strand cDNA Synthesis Kit (Thermo Scientific, Waltham, MA, USA) according to the manufacturer's instructions. Data were analyzed using the delta-delta Ct method and normalized to the housekeeping gene GAPDH. Each PCR run included a no-template control. Results are expressed as fold change from control. Specific primer sets used are as follows (5′ to 3′): 1*α*-hydroxylase, AAC CCT GAA CAA CGT AGT CTG CGA (forward) and ATG GTC AAC AGC GTG GAC ACA AA (reverse); VDR, CTG CTG AAG TCA AGT GCC AT (forward) and ACA AGT ACC GCG TCA GTG AC (reverse); tumor necrosis factor-*α* (TNF-*α*), CTG GGA TTC AGG AAT GTG TG (forward) and TTG ATC CCT GAC ATC TGG AA (reverse); interleukin (IL)-1*β*, CCA TGC AAT TTG TGT CTT CC (forward) and AGC CTG GAC TTT CCT GTT GT (reverse); IL-6, CTG CGC AGC TTT AAG GAG TT (forward) and TCC ACT GGG CAC AGA ACT TA (reverse); IL-8, ATC TGG CAA CCC TAG TCT GC (forward) and GTG AGG ACA TGT GGA AGC AC (reverse). All primers were synthesized by GenScript (Nanjing, China).

### 2.6. Quantitative Determination of 1,25D_3_

1,25D_3_ was quantified using an enzyme immunoassay kit for 1,25D_3_ (Immunodiagnostic Systems, Boldon, UK) according to the manufacturer's instructions.

### 2.7. Enzyme Linked Immunosorbent Assay (ELISA)

Cell-free supernatants were collected and aliquots were stored at −70°C until use. The protein levels of TNF-*α* and IL-8 in medium were measured with commercially available cytokine specific ELISA kits (R&D Systems, Minneapolis, MN, USA) according to the manufacturers' recommendations.

### 2.8. RNA Interference Experiments

16HBE cells were transfected with small interfering RNAs (siRNAs; GenePharma, Shanghai, China) against 1*α*-hydroxylase or VDR when reaching 70% confluence in six-well plates. A nonspecific siRNA (control siRNA) was used in each experiment as a negative control. For each transfection, 100 pmol siRNA per well was diluted in 250 *μ*L serum-free Opti-MEM I medium (Invitrogen Gibco) and gently mixed with 5 *μ*L Lipofectamine 2000 (Invitrogen Gibco) diluted in 250 *μ*L Opti-MEM I medium. After incubation for 25 min at room temperature, siRNA and Lipofectamine 2000 complexes were added to each well of six-well culture plates. The plates were gently mixed by rocking back and forth. Transfected cells were incubated for 24 h before being stimulated with* A. fumigatus*. Cell treatments are detailed in the figure legends.

### 2.9. Statistical Analysis

Data were expressed as either a representative experiment or the mean ± standard error (SE) of three independent experiments. Except where otherwise indicated, Student's paired *t*-test was used to compare the difference between groups, with *P* values < 0.05 considered statistically significant.

## 3. Results

### 3.1. *A. fumigatus* Induces the Expression of 1*α*-Hydroxylase and VDR and the Conversion of 25D_3_ to 1,25D_3_ in 16HBE Cells

**Figure 1:**
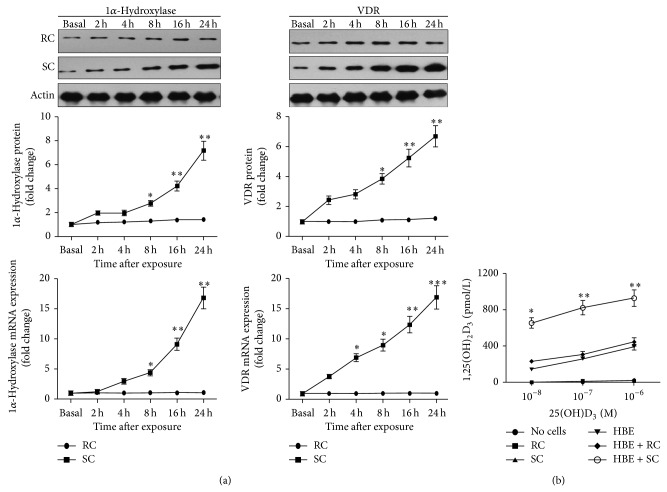
*A. fumigatus* induces the expression of 1*α*-hydroxylase and VDR and the conversion of 25D_3_ to 1,25D_3_ in 16HBE cells. (a) 16HBE cells were stimulated with resting conidia (RC) or swollen conidia (SC) [multiplicity of infection (MOI) = 1-2] for 2, 4, 8, 16, and 24 h. Nontreated 16HBE cells (basal) were used as controls. After treatment, cells were harvested and the protein and mRNA expression of 1*α*-hydroxylase and VDR was evaluated by Western blot analysis and quantitative real-time PCR, respectively. The Western blots illustrated are from one representative experiment of three separate ones, which are converted to densitometry units in graphs shown. 1*α*-Hydroxylase and VDR protein and mRNA expression was significantly upregulated in a time-dependent manner and peaked at 24 h after treatment with SC, whereas cells stimulated with RC showed no upregulation of 1*α*-hydroxylase and VDR protein and mRNA expression. All experiments were performed in triplicate on three consecutive days. Data shown are mean ± SE. Student's *t*-test, ^∗^ *P* < 0.05, ^∗∗^ *P* < 0.01, and ^∗∗∗^ *P* < 0.001, for comparison with baseline. (b) 16HBE cells were stimulated with RC or SC (MOI = 1-2) for 24 h in the presence of increasing doses of inactive vitamin D (25D_3_), and active vitamin D (1,25D_3_) was measured by ELISA in supernatants 24 h later. 16HBE cells converted the inactive vitamin D to the active form without other stimuli. 16HBE cells stimulated with SC generated greater amounts of active vitamin D in the presence of inactive vitamin D than cells without any treatment. RC stimulation did not influence the synthesis of active vitamin D in 16HBE cells. The graph reflects mean 1,25D_3_ levels and SEM of three independent experiments. Student's *t*-test, ^∗^ *P* < 0.05 and ^∗∗^ *P* < 0.01, for comparison of SC challenged cells with cells without any treatment.

To explore whether* A. fumigatus* has any effect on the expression of 1*α*-hydroxylase and VDR in 16HBE cells, we stimulated 16HBE cells with RC and SC. We observed basal expression of 1*α*-hydroxylase and VDR in nontreated 16HBE cells at both the protein and mRNA levels (Figure 1(a)). In cells stimulated with RC, protein and mRNA expression of 1*α*-hydroxylase and VDR remained at basal levels (Figure 1(a)). When challenged with SC, protein and mRNA expression of 1*α*-hydroxylase and VDR significantly increased in a time-dependent manner, peaking at 24 h after stimulation (Figure 1(a)).

We then examined whether* A. fumigatus* affects the conversion of inactive vitamin D to its active form. 16HBE cells stimulated with* A. fumigatus* were treated with increasing concentrations of inactive vitamin D (25D_3_), and the levels of active vitamin D (1,25D_3_) in supernatants were measured 24 h later (Figure 1(b)). We found that 16HBE cells could convert the inactive vitamin D to the active form in a dose-dependent manner without other stimuli. Consistent with the increased expression of 1*α*-hydroxylase, 16HBE cells stimulated with SC generated greater amounts of 1,25D_3_ than cells without any treatment when exposed to inactive vitamin D. No differences were found in the amounts of 1,25D_3_ in supernatants between nontreated cells and cells stimulated with RC in the presence of inactive vitamin D. These data were in agreement with the unchanged expression of 1*α*-hydroxylase in 16HBE cells challenged with RC. When inactive vitamin D was added to medium without cells, no active vitamin D was detected.

### 3.2. Locally Activated Vitamin D Synergistically Increases the Expression of LL-37 and HBD2 in 16HBE Cells Infected with* A. fumigatus* but Attenuates* A. fumigatus*-Induced Production of Chemokines and Cytokines

**Figure 2:**
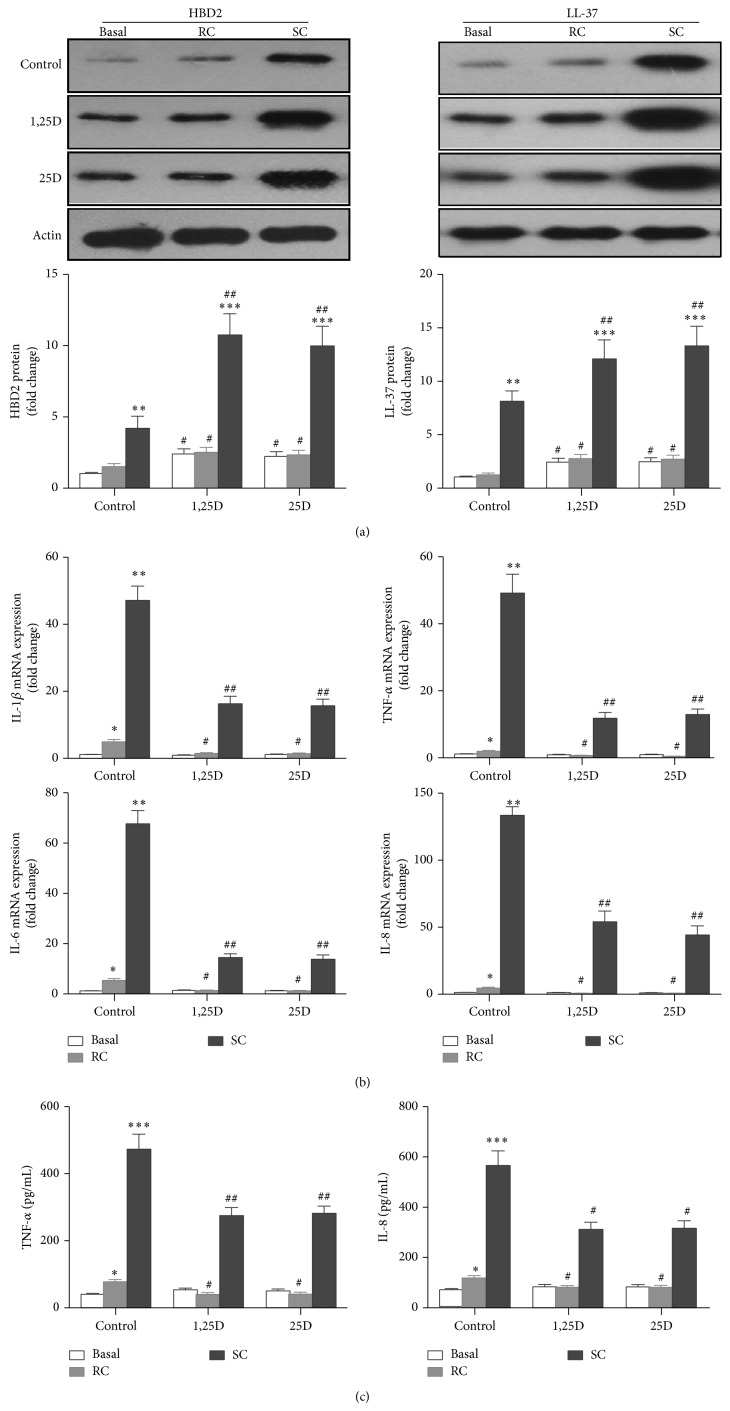
Locally activated vitamin D synergistically increases the expression of LL-37 and HBD2 in 16HBE cells infected with* A. fumigatus* but attenuates* A. fumigatus*-induced production of chemokines and cytokines. (a)–(c) 16HBE cells were untreated (basal) or stimulated with resting conidia (RC) or swollen conidia (SC) (MOI = 1-2) for 24 h in the presence of either inactive (25D_3_; 10^−7^ M) or active vitamin D (1,25D_3_; 10^−7^ M). Locally activated vitamin D synergistically induces protein expression of LL-37 and *β*-defensin-2 (HBD2) to a similar extent as exogenous active vitamin D (a). The protein expression of LL-37 and *β*-defensin-2 (HBD2) was evaluated by Western blot analysis. The Western blots illustrated are from one representative experiment out of three and converted to densitometry units in respective graphs. (b)-(c) Locally activated vitamin D attenuates* A. fumigatus*-induced production of chemokines and cytokines in 16HBE cells to a similar extent as exogenous active vitamin D. TNF-*α*, IL-1*β*, IL-6, and IL-8 mRNA expression was measured using quantitative real-time PCR (b). The concentrations of TNF-*α* and IL-8 in culture supernatants of 16HBE cells were measured by ELISA (c). Values reflect mean fold change from control and SEM of three independent experiments. Student's *t*-test, ^∗^ *P* < 0.05, ^∗∗^ *P* < 0.01, and ^∗∗∗^ *P* < 0.001, for comparison with baseline; ^#^ *P* < 0.05 and ^##^ *P* < 0.01, for comparison with control.

Vitamin D plays an important role in innate immunity. The active form of vitamin D has been proven to prevent infections by bacteria (e.g.,* M. tuberculosis*) or viruses by inducing the expression of the cathelicidin antimicrobial peptide and *β*-defensins [44, 45] and decreasing the inflammatory response to microbial infections [16, 46–49]. Exposure of respiratory epithelial cells to* A. fumigatus* increases the release of LL-37 and *β*-defensins and the expression of chemokines and cytokines that initiate an inflammatory reaction [3, 5–7]. Having established that 16HBE cells generate more active vitamin D and have upregulated VDR expression after stimulation with* A. fumigatus*, we hypothesized that when exposed to the inactive form of vitamin D, 16HBE cells stimulated with* A. fumigatus* would convert it to 1,25D_3_, which would alter the expression of inflammatory mediators and antimicrobial peptides induced by* A. fumigatus* in an autocrine fashion. To test this hypothesis, we initially treated 16HBE cells with 10^−7^ M of either the active or inactive form of vitamin D (1,25D_3_ or 25D_3_) and examined the protein expression of LL-37 and HBD2 in 16HBE cells stimulated with* A. fumigatus* for 24 h (Figure 2(a)). We found that both forms of vitamin D enhanced the basal expression of LL-37 and HBD2 in 16HBE cells to a similar extent. Regardless of treatment with either form of vitamin D, stimulation with RC did not change the expression of LL-37 or HBD2 in 16HBE cells. When stimulated with SC, the expression of LL-37 and HBD2 increased significantly. The presence of either 1,25D_3_ or 25D_3_ significantly augmented SC-induced expression of LL-37 and HBD2 to a similar extent in 16HBE cells.

Subsequently, we sought to examine whether locally activated vitamin D affects* A. fumigatus*-induced production of TNF-*α*, IL-1*β*, IL-6, and IL-8 in 16HBE cells. We found significant increases in the mRNA expression of TNF-*α*, IL-1*β*, IL-6, and IL-8 after stimulation with both RC and SC for 24 h (Figure 2(b)), an effect that was more profound with SC stimulation. Treatment with either 1,25D_3_ or 25D_3_ significantly inhibited SC- and RC-induced upregulation of all these inflammatory mediators in 16HBE cells to a similar extent (Figure 2(b)). We did not find any changes in the mRNA expression of TNF-*α*, IL-1*β*, IL-6, and IL-8 when 16HBE cells were exposed to either form of vitamin D alone (Figure 2(b)). We also measured the concentrations of TNF-*α* and IL-8 in culture supernatants of 16HBE cells using the appropriate ELISA (Figure 2(c)). Results showed that the protein expression of TNF-*α* and IL-8 induced by* A. fumigatus* was significantly attenuated in the presence of both forms of vitamin D.

Taken together, these data show that 1,25D_3_ generated by 16HBE cells is indeed biologically active and regulates the antifungal immune responses of 16HBE cells to* A. fumigatus* to a similar extent as exogenous exposure to 1,25D_3_. Higher expression of LL-37 and HBD2 enhanced by vitamin D may have the potential of locally enhancing the innate immunity within the lungs. The inhibited expression of inflammatory mediators may avoid tissue damage caused by excessive inflammation.

### 3.3. Silencing of VDR or 1*α*-Hydroxylase Attenuates the Effects of Locally Activated Vitamin D on the Response of 16HBE Cells to* A. fumigatus*

**Figure 3:**
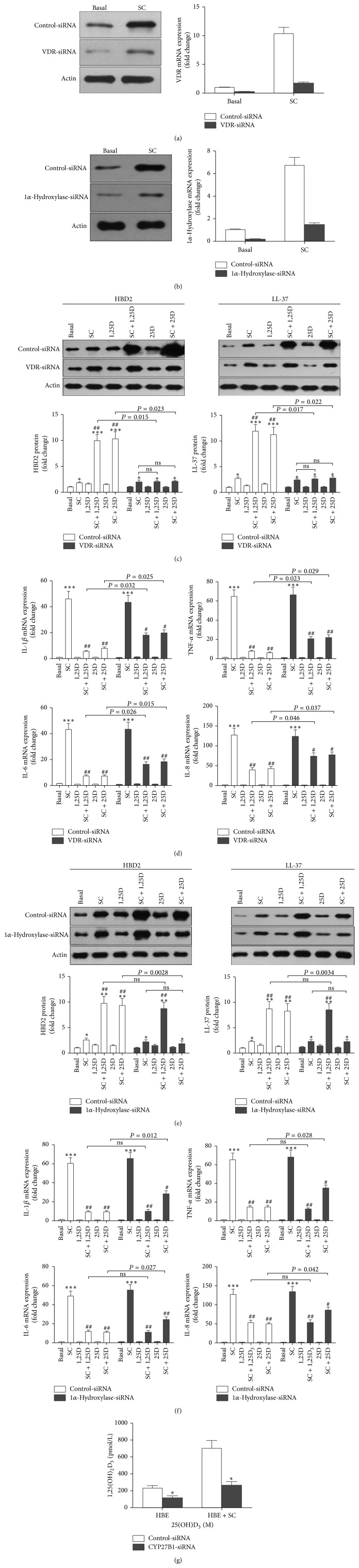
Silencing VDR or 1*α*-hydroxylase attenuates the effects of locally activated vitamin D on the responses of 16HBE cells to* A. fumigatus*. (a), (b) 16HBE cells were transfected with a control siRNA or an siRNA against VDR or 1*α*-hydroxylase for 24 h and then challenged with SC (MOI = 1-2) for another 24 h. The protein and mRNA levels of VDR (a) and 1*α*-hydroxylase (b) were measured by Western blot analysis and quantitative real-time PCR, respectively. (c)–(f) 16HBE cells were transfected with a control siRNA or an siRNA against VDR or 1*α*-hydroxylase for 24 h and then stimulated with SC (MOI = 1-2) for 24 h in the presence of either inactive (25D_3_; 10^−7^ M) or active vitamin D (1,25D_3_; 10^−7^ M). The protein expression of LL-37 and HBD2 was evaluated by Western blot analysis. The Western blots illustrated are from one representative experiment out of three, which are converted to densitometry units in respective graphs. The mRNA expression of TNF-*α*, IL-1*β*, IL-6, and IL-8 was evaluated by quantitative real-time PCR. When VDR expression was silenced, the modulatory effects of both forms of vitamin D on LL-37, HBD2, TNF-*α*, IL-1*β*, IL-6, and IL-8 expression in 16HBE cells induced by SC were reduced ((c), (d)); when 1*α*-hydroxylase expression was silenced, only the effect of 25D_3_ was reduced ((e), (f)). Values reflect mean fold change from control and SEM of three independent experiments. Student's *t*-test, ^∗^ *P* < 0.05, ^∗∗^ *P* < 0.01, and ^∗∗∗^ *P* < 0.001, for comparison with baseline; ^#^ *P* < 0.05 and ^##^ *P* < 0.01, for comparison with SC. (g) CYP27B1 silencing reduces the synthesis of active vitamin D induced by SC in 16HBE cells. 16HBE cells were transfected with a control siRNA or an siRNA against 1*α*-hydroxylase for 24 h and then stimulated with SC (MOI = 1-2) for 24 h in the presence of inactive vitamin D (25D_3_; 10^−7^ M); active vitamin D (1,25D_3_) in supernatants was measured by ELISA. Graph reflects mean 1,25D_3_ levels and SEM of three independent experiments. Student's *t*-test, ^∗^ *P* < 0.05, for comparison with control siRNA transfected cells.

To definitively link 1,25D_3_ generation to SC-induced 1*α*-hydroxylase expression in 16HBE cells and to confirm that the local activation of vitamin D directly mediates the responses of 16HBE cells to stimulation with* A. fumigatus* in an autocrine manner, we next explored whether silencing of 1*α*-hydroxylase or VDR would reverse the effects of locally activated vitamin D on the response of 16HBE cells to* A. fumigatus*. We performed knockdown experiments using siRNAs against 1*α*-hydroxylase or VDR. Knockdown of the 1*α*-hydroxylase or VDR gene was confirmed by Western blot and quantitative real-time analyses (Figures 3(a) and 3(b)). When cells were transfected with the control siRNA, both forms of vitamin D synergistically enhanced SC-induced upregulation of LL-37 and HBD2 expression and reduced SC-induced expression of TNF-*α*, IL-1*β*, IL-6, and IL-8, as shown previously in nontransfected cells (Figures 3(c)–3(f)). When VDR expression was silenced, both forms of vitamin D no longer had any modulatory effect on LL-37 and HBD2 (Figures 3(c) and 3(e)), and their inhibition of chemokines and cytokines induced by SC was moderately, but significantly, impaired (Figure 3(d)). However, when 1*α*-hydroxylase expression was silenced, only the effect of 25D_3_ was eliminated (Figures 3(e) and 3(f)). Moreover, silencing of 1*α*-hydroxylase significantly reduced its ability to convert 25D_3_ to 1,25D_3_ in 16HBE cells challenged with SC (Figure 3(g)). This finding further supports our primary hypothesis that when exposed to the inactive form of vitamin D, 16HBE cells stimulated with* A. fumigatus* would convert it to 1,25D_3_, further altering the expression of inflammatory mediators and antimicrobial peptides in an autocrine fashion.

### 3.4. *β*-Glucan, an* A. fumigatus* Wall Component, Increases the Expression of 1*α*-Hydroxylase and VDR, and Vitamin D Synergistically Increases *β*-Glucan-Induced Expression of Antimicrobial Peptides but Attenuates *β*-Glucan-Induced Expression of Chemokines and Cytokines in 16HBE Cells

**Figure 4:**
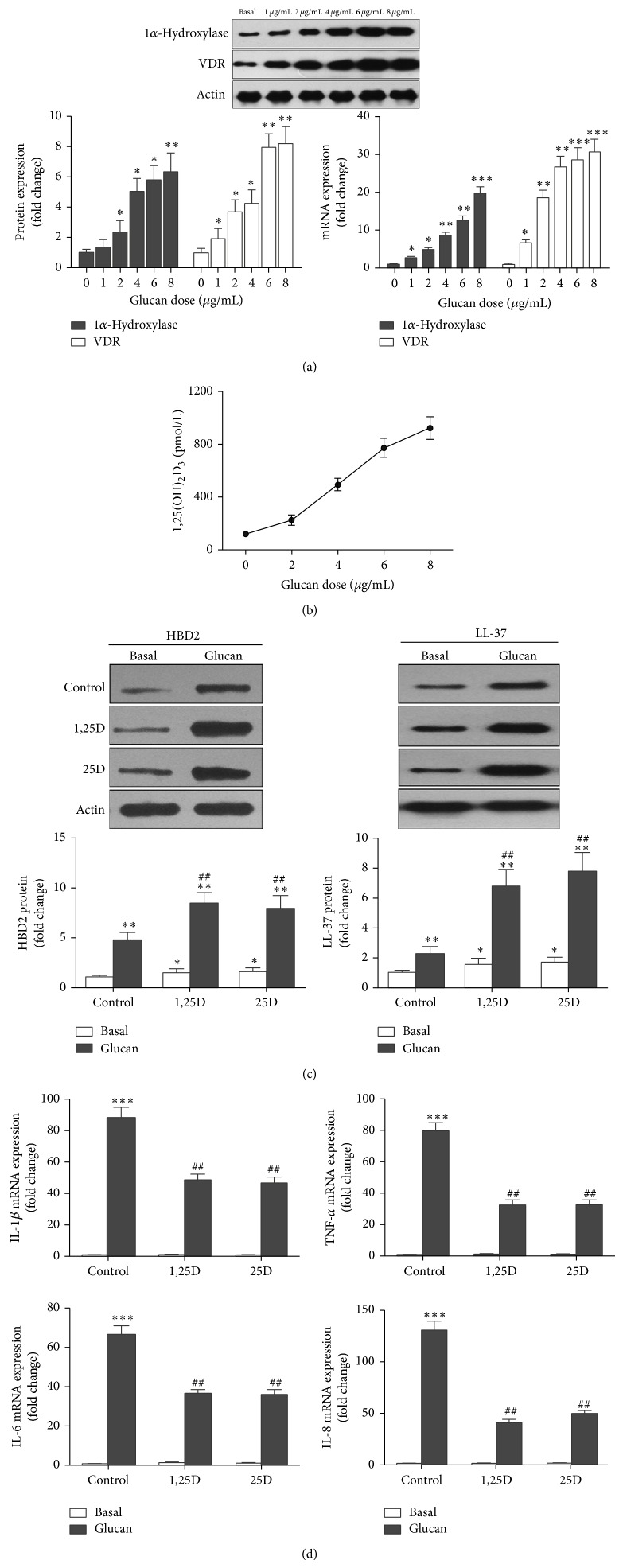
*β*-Glucan increases the expression of 1*α*-hydroxylase and VDR, and vitamin D synergizes with *β*-glucan to induce the expression of antimicrobial peptides but attenuates *β*-glucan-induced expression of chemokines and cytokines in 16HBE cells. (a) *β*-Glucan increases the expression of 1*α*-hydroxylase and VDR in 16HBE cells in a dose-dependent manner. 16HBE cells were cultured in the absence or presence of increasing doses of *β*-glucan as indicated for 24 h. Cells were then harvested and the protein and mRNA expression of 1*α*-hydroxylase and VDR was evaluated by Western blot analysis and quantitative real-time PCR, respectively. The Western blots shown are from one representative experiment out of three, which are converted to densitometry units in graphs. Values reflect mean fold change from control and SEM of three independent experiments. Student's *t*-test, ^∗^ *P* < 0.05, ^∗∗^ *P* < 0.01, and ^∗∗∗^ *P* < 0.001, for comparison with baseline. (b) *β*-Glucan increases the conversion of inactive vitamin D (25D_3_) to active vitamin D (1,25D_3_) in a dose-dependent manner. 16HBE cells were cultured in the presence of increasing doses of *β*-glucan as indicated and inactive vitamin D (10^−7^ M) for 24 h, and active vitamin D (1,25D_3_) in supernatants was measured by ELISA. Graph reflects mean 1,25D_3_ levels and SEM of three independent experiments. (c), (d) 16HBE cells were cultured in the absence or presence of *β*-glucan (8 *μ*g/mL) with or without either form of vitamin D (10^−7 ^M, 25D, and 1,25D) for 24 h. Cells were harvested and the protein expression of LL-37 and HBD2 was evaluated by Western blot analysis. The Western blots illustrated are from one representative experiment out of three, which are converted to densitometry units in graphs. The mRNA expression of TNF-*α*, IL-1*β*, IL-6, and IL-8 was evaluated by quantitative real-time PCR. Vitamin D synergizes with *β*-glucan to induce the expression of LL-37 and HBD2 (c) but attenuates *β*-glucan-induced expression of TNF-*α*, IL-1*β*, IL-6, and IL-8 in 16HBE cells (d). Values reflect mean fold change from control and SEM of three independent experiments. Student's *t*-test, ^∗^ *P* < 0.05, ^∗∗^ *P* < 0.01, and ^∗∗∗^ *P* < 0.001, for comparison with baseline; ^#^ *P* < 0.05 and ^##^ *P* < 0.01, for comparison with control.

The cell walls of fungi, including* A. fumigatus,* are in a large part made up of *β*-glucan. We thus examined whether *β*-glucan has any effect on the expression of 1*α*-hydroxylase and VDR in 16HBE cells. We stimulated 16HBE cells with *β*-glucan for 24 h and examined the expression of 1*α*-hydroxylase and VDR at both protein and mRNA level. Results showed that *β*-glucan significantly increased the expression of 1*α*-hydroxylase and VDR in a dose-dependent manner (Figure 4(a)). Furthermore, 16HBE cells converted more 25D_3_ to 1,25D_3_ when *β*-glucan was present (Figure 4(b)). We then investigated the protein expression of LL-37 and HBD2 and found that *β*-glucan could induce LL-37 and HBD2 expression by itself. 16HBE cells exposed to *β*-glucan in the presence of inactive or active vitamin D had significantly greater amplification of LL-37 and HBD2 expression than by *β*-glucan alone (Figure 4(c)). In contrast to the synergistic effect of vitamin D exposure and *β*-glucan on LL-37 and HBD2 expression, both forms of vitamin D decreased *β*-glucan-induced expression of TNF-*α*, IL-1*β*, IL-6, and IL-8 at the mRNA level (Figure 4(d)). These data suggest that *β*-glucan induces vitamin D conversion and recognition in airway epithelial cells and that vitamin D synergizes with *β*-glucan to induce expression of antimicrobial proteins but attenuates *β*-glucan-induced expression of chemokines and cytokines in airway epithelial cells.

## 4. Discussion

Our results showed that* A. fumigatus* SC induced 1*α*-hydroxylase and VDR expression in 16HBE cells and led to increased local activation of vitamin D. Locally activated vitamin D could synergize with SC to further amplify the expression of LL-37 and HBD2 in 16HBE cells but attenuate SC-induced production of chemokines and cytokines in an autocrine fashion. Furthermore, we identified *β*-glucan, the major component of the* A. fumigatus* cell wall, as the causative agent responsible for upregulation of 1*α*-hydroxylase and VDR expression in 16HBE cells.

In this study,* A. fumigatus* RC were also used for the stimulation of 16HBE cells, but no induction of 1*α*-hydroxylase or VDR was observed. RC are surrounded by a hydrophobic layer of rodlet proteins [42]. The rodlet layer could protect conidia from recognition and phagocytosis by the host defense system. Conidial swelling releases the protective rodlet layer and exposes *β*-glucan [42]. Therefore, exposed *β*-glucan might be responsible for mediating the effect that* A. fumigatus* impacts vitamin D activation and signal transduction and thus affects the antifungal immunity in the airway epithelium. Our finding that *β*-glucan increased the expression of 1*α*-hydroxylase and VDR in 16HBE cells further confirmed this presumption. In human macrophages, toll-like receptor 2/1 (TLR2/1) and TLR8 activation upregulates the expression of VDR and 1*α*-hydroxylase [50, 51]. In the human skin, 1*α*-hydroxylase expression was increased after being wounded [25]. TLR2/6 ligands were also found to increase 1*α*-hydroxylase expression in cultured keratinocytes [25]. In human respiratory epithelial cells, respiratory syncytial virus (RSV) and Poly(IC), a synthetic analog of dsRNA, but not bacterial cell wall components (TLR2 ligands), were observed to increase the expression of 1*α*-hydroxylase [19]. The factors associated with regulation of 1*α*-hydroxylase and VDR expression in barrier tissues are less clear. Whether this phenomenon is common when pathogenic microorganisms are recognized by cells at the barrier sites is still unknown, awaiting further exploration.

Our results are different from those reported by Coughlan et al. who found that* A. fumigatus* downregulated VDR expression in airway epithelial cells and identified gliotoxin as the causative agent responsible for mediating this effect [52]. This obvious discrepancy between the two studies could be explained by the use of* A. fumigatus* at different growth stages. During asexual growth, the morphological form of* A. fumigatus* changes from RC to SC which then forms germ tubes that continue growing in hyphal form. The surfaces of various* A. fumigatus* morphotypes differ from each other and, consequently, the reaction of host cells may vary towards different* A. fumigatus* growth forms [53, 54]. Our previous study has shown that in the lungs of immunocompetent hosts* A. fumigatus* could hardly grow into hyphae [40]. So in the present study, we used SC for stimulation, in which *β*-glucan was one of the major antigens to be recognized by the airway epithelium. In order to find out whether* Aspergillus* colonization in the lungs of ABPA patients affect VDR expression in airway epithelial cells, Coughlan et al. stimulated these cells with culture filtrates from* A. fumigatus* grown for 4 days, when the hyphae of* A. fumigatus* form [52]. Gliotoxin is secreted by the hyphal form of* A. fumigatus* [52, 55], so hypha formation might compromise the positive impact conferred by SC-induced vitamin D synthesis and VDR expression in the airway epithelium. Our findings are an important supplement to the findings reported by Coughlan et al. These two completely different results also indicate host morphotype's specific reactions to* A. fumigatus.*

Vitamin D synergizes with both SC and *β*-glucan to further amplify the expression of the two antimicrobial proteins, LL-37 and HBD2, which have antifungal activity [56, 57]. And defensins have additional activities such as the chemoattraction of immature dendritic cells, T cells, and monocytes as well as activation of the professional antigen-presenting cells [58–60]. Conversely, vitamin D attenuated the induction of proinflammatory cytokines and the chemokine IL-8 by* A. fumigatus* and *β*-glucan. Microbial infection causes cytokine-associated inflammation to remove pathogens. However, excessive or uncontrolled release of proinflammatory cytokines and chemokines can damage the integrity of gas-exchange surfaces, even cause immunodeficiency, septic shock, or induction of autoimmunity, and eventually impair disease eradication [9, 61–63]. It has been proposed that in fungal infections disease pathology may be attributable to an aggravated or dysregulated host inflammatory response that results in extensive tissue damage. In mice with chronic granulomatous disease, the intrinsic, genetically determined failure to control inflammation after exposure to sterile fungal components determined the animals' inability to resolve an infection with* A. fumigatus* [64]. In neutropenic patients with aspergillosis, the clinical and radiologic pulmonary deterioration during neutrophil recovery may be aspergillosis-related immune reconstitution inflammatory syndrome [65]. Thus, local synthesis of active vitamin D might be one of the ways by which the airway epithelium enhances its antifungal capability while limiting inflammation-induced tissue damage in challenge with* A. fumigatus*, suggesting the potential beneficial effects of proper 25D_3_ levels in blood circulation on pathogen-driven inflammation in respiratory epithelium.

In conclusion, we have shown that* A. fumigatus* led to more significant local activation of vitamin D, which resulted in enhanced antimicrobial peptide production and attenuating cytokine release in respiratory epithelial cells. We confirmed locally enhanced innate immunity and mitigated inflammation after exposure of airway epithelial cells stimulated with* A. fumigatus* to the inactive vitamin D. Hence, vitamin D might provide a novel treatment option that may reduce lung inflammation and disease severity in* Aspergillus* infection, without negatively affecting* Aspergillus* clearance. Given that low serum 25D_3_ levels are prevalent and have been associated with increased incidences of acute respiratory symptoms and lower respiratory infection [29, 36, 37], supplemental administration of vitamin D might be advocated for its potential to prevent* A. fumigatus* infection. It will be important to investigate whether disturbed vitamin D metabolism contributes to the pathopoiesis of* A. fumigatus* and whether local activation of vitamin D could enhance the barrier function of airway epithelium and delay the hyphal formation of* A. fumigatus*. Our findings may provide valuable insight into the role of vitamin D in susceptibility to pulmonary aspergillosis in immunosuppressed patients.
